# Hypoglycemic and Hypotensive Effects of *Calycotome villosa* Subsp. *intermedia* Seeds in *Meriones shawi* Rats: In Vivo, Ex Vivo, and In Vitro Investigations

**DOI:** 10.1155/2023/3081102

**Published:** 2023-05-11

**Authors:** Badiaa Lyoussi, Hassan Laaroussi, Khadija Cherkaoui-Tangi, Christophe Hano, Nicole Morel

**Affiliations:** ^1^Laboratory of Natural Substances, Pharmacology, Environment, Modeling, Health and Quality of Life (SNAMOPEQ), Department of Biology, Faculty of Sciences Dhar Mehraz, Sidi Mohamed Ben Abdallah University, 30000 Fez, Morocco; ^2^Secteur des Sciences de la Santé, Université Catholique de Louvain, Bruxelles, Belgium; ^3^Department of Biological Chemistry, University of Orleans, Eure et Loir Campus, Chartres, France

## Abstract

*Calycotome villosa subsp. intermedia* is used in traditional medicine for the prevention and self-treatment of a variety of illnesses, including diabetes mellitus, obesity, and hypertension. The present study aims to investigate the *in vivo*, *ex vivo*, and *in vitro* hypoglycemic and hypotensive effects of the lyophilized aqueous extract of *Calycotome villosa subsp*. *intermedia* seeds (CV) on *Meriones shawi* submitted to hypercaloric diet and physical inactivity (HCD/PI) for 12 weeks. This diet induces a type 2 diabetes/metabolic syndrome phenotype with hypertension. Furthermore, HCD/PI decreased aorta contraction due to noradrenaline, enhanced L-arginine, and depressed insulin-evoked relaxation, while the relaxing effects of the NO donor SNAP and of diazoxide were unchanged. *In vivo* experiments showed that the oral administration of the CV extract (50 mg/kg b.wt) for 3 consecutive weeks significantly attenuated the development of type 2 diabetes, obesity, dyslipidemia, and hypertension. These effects may involve the improvement of lipid metabolism, insulin sensitivity, systolic arterial pressure, and urine output. Additionally, *ex vivo* and *in vitro* investigations revealed that CV treatment improved vascular contraction to noradrenaline, induced a slight aorta relaxation in response to carbachol, increased the vasorelaxation effect evoked by insulin, and depressed the L-arginine evoked relaxation. However, CV did not change the endothelium-independent vasorelaxation response evoked by SNAP or diazoxide. Hence, the present study provides useful information and supports the traditional use of CV in the prevention and self-treatment of numerous ailments. Overall, it can be concluded that *Calycotome villosa subsp*. *intermedia* seed extracts might be useful in the management of type 2 diabetes and hypertension.

## 1. Introduction

Metabolic syndrome (Mets) is a complex pathophysiologic state characterized by obesity, dyslipidemia, insulin resistance, and hypertension. These metabolic disorders are often associated with oxidative stress, chronic inflammation, endothelial dysfunction, and vascular dysregulation leading to cardiometabolic and cardiovascular diseases [1]. It is well known that Mets development is positively linked with several bad habits and risk factors such as alcohol consumption, smoking, physical inactivity, and low cardiorespiratory fitness [2].

Over the past 15 years, these diseases and their serious complications have remained the prominent cause of death worldwide [3]. Since adipose tissue plays a key role in regulating glucose homeostasis and energy metabolism, obesity is most often linked with an increased risk of developing insulin resistance (IR). In obesity conditions, adipose tissue releases high amounts of proinflammatory cytokines, glycerol, nonesterified fatty acids, and other adipocyte-bioactive mediators such as adiponectin and leptin that are involved in the development of insulin resistance and thus T2DM [4]. In addition to adipocyte-derived factors, the upregulation of monocyte chemoattractant protein-1 (MCP-1), tumor interleukin-6 (IL-6), and necrosis factor-*α* (TNF-*α*) might also have a role in the development of insulin resistance and promote the pathophysiological link between metabolic dysfunction and cardiovascular disease [5]. In return, uncontrolled chronic hyperglycemia associated with obesity may lead to serious CVD. The relationship between diabesity and cardiovascular diseases (CVD) has been already documented. In fact, diabesity is frequently associated with both microvascular and macrovascular complications and plays a pivotal role in the initiation of several cardiometabolic diseases through diverse mechanisms and signaling pathways, including (i) inflammation, (ii) overproduction of reactive oxygen species (ROS) and free radicals, and (iii) increased formation of endogenous advanced glycation end products (AGEs) [6].

Actually, conventional interventions and treatment strategies based on the single or combined use of chemical and biochemical antihyperglycemic and hypoglycemic agents, e.g., thiazolidinediones, sulphonylureas, metformin, sodium-glucose cotransporter-2 (SGLT-2) inhibitors, dipeptidyl peptidase-4 (DPP-IV) enzyme inhibitors, insulin, or glucagon-like peptide-1 (GLP-1) agonists are the mainstay of diabetes management [7]. However, the conventional treatment of diabetes and its serious complications requires a heavy and substantial economic cost for both developing and developed countries worldwide [8]. Moreover, chronic treatment using orthodox medicine may be associated with several undesirable side effects including gastrointestinal disturbances, abdominal pain, body weight gain, hypoglycemia, lactic acidosis, and nausea [9]. Therefore, research for new effective and safer antidiabetic agents is needed to regulate chronic hyperglycemia, hypertriglyceridemia, and associated CVD and Mets.

Recently, different strategies have been proposed to prevent and improve Mets including the promotion of a healthy lifestyle (physical activity, dietary rich antioxidants, low fat, and carbohydrate dairy product consumption).

Previous studies have documented the beneficial effect of rich-antioxidant Mediterranean dietary patterns (legumes, vegetables, fruits, whole seeds, olive oil, nuts, etc.) on diabetes, hypertension, and CVD [10, 11]. However, the search for new safer and bioactive compounds from functional foods and medicinal plants remains of great interest.

*Calycotome villosa* (Poiret) Link *subsp*. *intermedia* is a medicinal plant belonging to the *Papilionacea* family. It is an erect shrub that can grow up to 2 m tall, especially in the north of Africa and Spain [12]. This plant is still used in Moroccan folk medicine as an infusion to treat rheumatism and a variety of other diseases, such as wound scars and hepatic and renal dysfunctions [13]. Chrysin glucoside, an active component of CV, has been isolated previously by our research team and demonstrated to have diuretic, vasodilator, and hypotensive effects in *Meriones* rats [14]. Recently, Boughalleb and coworkers have reported that *Calycotome villosa* seeds contain several antioxidant compounds belonging to different chemical groups such as quercetin, rutin, kaempferol, luteolin, apigenin, naringenin, epicatechin, p‐coumaric acid, syringic acid, and quinic acid [15]. These compounds are also present in other medicinal plants and functional food products known for their antioxidative, antidiabetic, and antihypertensive properties [16, 17].

To the best of our knowledge, there is no record data on *in vitro* or *in vivo* hypoglycemic and hypotensive effects of CV extracts. Thus, the purpose of the present study was to investigate the hypoglycemic and hypotensive effects of the CV seed extract on diabetic and hypertensive *Meriones shawi* rats. The findings of the present investigation provide useful information and confirm the traditional use of CV extracts as a dietary supplement for the management of diabesity and associated metabolic disorders.

## 2. Materials and Methods

### 2.1. Plant Material and Extract Preparation

Seeds of *Calycotome villosa subsp*. *intermedia* were collected from the areal part of the plant from Sefrou province, Morocco. Authentic samples were identified at the Department of Biology, Faculty of Science, Sidi Mohamed Ben Abdellah University Fès, Morocco, where a voucher specimen has been deposited (LB134).

The *Calycotome villosa subsp*. *intermedia* seed extract was prepared as it is used traditionally in Morocco. The air-dried seeds were ground to a coarse powder in an electric grinder (Omni mixer 17106, Du Pont Company, USA). Then, 50 g of the powder was suspended in 1 L of distilled water and heated to boil under reflux for 30 min and cooled for 15 min. Thereafter, the obtained decoction (the aqueous extract) was centrifuged and filtered using a millipore filter (Millipore 0.2 mm, St. Quentin en Yvelines, France) to remove particulate matter. Subsequently, the filtrate was frozen at −20°C and then lyophilized (FreeZone® Dry 4.5, USA). The yield of the plant extract after lyophilization was 17.43%. Distilled water was added to prepare the chosen concentration (50 mg/kg b.wt).

### 2.2. Experimental Design

#### 2.2.1. Type 2 Diabetes Installation

Based on our previous study, to install the type 2 diabetes/metabolic syndrome phenotype with hypertension, adult male *Meriones shawi* rats (15–18 week-old) were fed a hypercaloric diet (612 kcal/100 g bwt/day) containing mainly 22.78% of proteins, 18.76% of complex carbohydrates, and 49.57% of fat and restrained to limited physical activity during 12 weeks [1].

Body weight, blood glucose, total cholesterol, and triglyceride were measured every 2 weeks during 12 weeks. Only rats with fasting blood glucose level (BGL) ≥200 mg/dL, total cholesterol (TC) ≥3.00 mmol/L, and triglyceride (TG) ≥1.3 mmol/L were selected and used in this study.

#### 2.2.2. Experimental Animal's Protocol

Thirty male *Meriones shawi* rats weighing 200.12 ± 6.34 g, living in the semiarid climate, were bred in the Department of Physiology, Faculty of Sciences Dhar El Mehraz, Fez, Morocco and housed in standard environmental conditions (22 ± 3°C, 55 ± 5% humidity, and 12 h light/dark cycles). Investigations using experimental animals were approved by the institutional research committee, and ethical approval was obtained from Sidi Mohamed Ben Abdellah University, Fez, under the responsibility of the Animal Facility and the Laboratory of Natural Substances, Pharmacology, Environment, Modeling, Health and Quality of Life, University of Sidi Mohamed Ben Abdellah, Fez, Morocco (USMBA-SNAMOPEQ 2013-03). Experiments were conducted following the internationally accepted principles for laboratory animal use and care for animals following the EU Directive 2010/63/EU for animal experiments to avoid and minimize animal suffering and the number of experimented animals.

After the T2D/metabolic syndrome phenotype with hypertension installation, *Meriones* were randomly allocated into 5 groups, with 6 rats in each group. Treatments and applied procedures are as follows:

  Group 1: HCD/SLS + DW, diabetic animals, received daily by gavage distilled water (10 mL/kg BW) and had free access to standard chow and tap water for 3 weeks;
  Group 2: NC + DW, nondiabetic rats, received daily by gavage distilled water (10 mL/kg BW) and had free access to tap water for 3 weeks;
  Group 3: NC + CV, nondiabetic rats, received daily by gavage *Calycotome villosa* Subsp. *Intermedia* seed extract (50 mg/kg BW) and had free access to tap water for 3 weeks;
  Group 4: HCD/SLS + CV, diabetic rats, received daily by gavage *Calycotome villosa* Subsp. *Intermedia* seed extract (50 mg/kg BW) for 3 weeks;
  Group 5: HCD/SLS+(Aml + *G*), diabetic rats, treated by amlodipine (1 mg/kg BW, i.p.) and glibenclamide (2.5 mg/kg BW, orally) for 3 weeks.

Rats of all groups received a normal chow diet (carbohydrate (48%), protein (21%), fat (3%), fiber (5%), calcium (0.8%), phosphorus (0.4%), moisture (13%), and ash (8%)) twice a day.

The treatment of animals started immediately on day 1 after the installation of T2D (12 weeks) which was considered the first day of treatment. At the end of the experiment period (3 weeks), fasted rats (12 hours after their last feeding) were anesthetized with light diethyl ether. Blood samples were collected from each rat by retroorbital bleeding into EDTA tubes, and then plasma was separated by centrifugation (2000 × g) for 10 min. For the collection of urine samples, rats were kept individually in specific metabolic cages, and urine of 24 h were collected. *Meriones* had free access to normal chow diet and tap water during the urine collection period (24 h).

### 2.3. Oral Glucose Tolerance Test

After 18 hours of fasting, normal and treated *Meriones* were given 1.5 g/kg of glucose orally. DW, CV, and Aml + *G* were administered to rats in their respective groups 30 minutes later after glucose administration. Blood samples were collected from the caudal vein at −30, 0, 15, 30, 45, 60, 120, and 180 minutes after glucose administration, and then the glucose levels were estimated using the Accu-Chek glucometer.

### 2.4. Biochemical Analysis

After the treatment duration (3 weeks), blood and urinary samples were collected for the analysis of blood glucose concentration (Accu-Chek glucometer, Roche), total cholesterol (TC) (kit number 7D62-20 and cholesterol oxidase/POD method), triglycerides (TG) (kit number 7D74-20 and lipase/GK/POD method), urea (kit number 7D75-30 and urease/NADH method), and creatinine (kit number 7D64-20 and picric acid/NaOH method). Urinary sodium (Na^+^) (kit number LN9D28-02), potassium (K^+^) (kit number 1E48-20), and chloride (Cl^−^) (kit number 1E49-01) were analyzed using the ion-selective potentiometry method (Architect c8000i biochemistry analyzer) [17, 18].

### 2.5. Assessment of Vascular Function

Vascular effects of the CV extract were tested *ex vivo* on aorta isolated from *Meriones* of all groups as detailed in our previous investigations [1, 16]. *Meriones* of all groups were anesthetized with diethyl ether and sacrificed by decapitation. Then, thoracic aortas were immediately isolated and cleaned of excess fat and adherent connective tissues and cut into rings of about 2 mm length. Each individual segment was suspended between two hooks and mounted in 12.5 ml organ baths filled with a physiological solution of the following composition (mM): NaCl, 122; KCl, 5.9; NaHCO_3_, 15; MgCl_2_, 1.25; CaCl_2_, 1.25; glucose, 11; and supplemented with indomethacin (10 *μ*M) and aerated with 95% O_2_ and 5% CO_2_. A basal tension of 20 mN was applied to the artery rings. After an equilibration period, each preparation was contracted by changing the physiological solution in the bath to a depolarizing 100 mM KCl solution (composition in mM: NaCl, 27; KCl, 100; NaHCO_3_, 15; MgCl_2_, 1.25; CaCl_2_, 1.25; and glucose, 11).

Endothelium integrity was tested by measuring the relaxation evoked by carbachol (1 *μ*M). After washing and 60 min recuperation time, test contraction was evoked either by changing the solution in the bath to the KCl solution or by adding noradrenaline to the physiological solution in the bath. For the relaxation studies, the aortic rings were precontracted with noradrenaline (1 *μ*M). When noradrenaline-induced contraction had reached a plateau level, carbachol (10 nM–10 *μ*M), sodium nitroprusside (10 nM–0.3 *μ*M), or diazoxide (10 nM–30 *μ*M) were added in the bath.

To investigate the effect of NO synthase inhibition on vascular reactivity, aortic rings were incubated with N-nitro-L-arginine (L-NOArg, 100 *μ*M) for 30 min before a noradrenaline-induced contractile response was evoked.

At the end of each experiment, tissues were blotted on a filter paper using a roller and weighed. The amplitude of contraction was normalized to the weight of the artery ring. Relaxation was expressed as a percentage of precontracted tension.

## 3. Results

### 3.1. Pathology Development in *Meriones shawi* Rats: Biometabolic Parameters

The present data indicate that normal *Meriones shawi* rats submitted to a high-calorie diet (HCD) (612 kcal/day/100 g body weight) associated with a sedentary lifestyle (SLS) (physical inactivity) for 12 weeks (see Table 1) expressed a significant elevation of fasting blood glucose (222.00 vs. 76.00 mg/dL), total cholesterol (3.19 vs. 1.21 mmol/L), plasma triglycerides (1.39 vs. 0.58 mmol/L) levels as compared to the baseline (day 0), and final body weight (219 vs. 103 g) (*p* < 0.001). TC was the first parameter statistically changed followed by body weight, TG, and finally blood glucose.

### 3.2. *In Vivo* Effects of *Calycotome villosa subsp. intermedia* Seed Extracts

#### 3.2.1. Blood Glucose Level Changes during 180 minutes

As illustrated in Table 2, diabetic animals receiving a single dose (50 mg/.bwt) of the CV extract (HCD/SLS + CV) showed a significant reduction (*p*  < 0.001) of blood glucose levels (BGL) at 180 minutes after treatment compared to nontreated diabetic animals (HCD/SLS + DW) and BGL at 0 minute. However, the administration of a single dose of the CV extract to normal *Meriones shawi* rats (NC + CV) did not change the normal BGL during all time intervals.

#### 3.2.2. Body Weight, Fasting Blood Glucose, Heartbeat, and Systolic Arterial Pressure

As shown in Table 3, feeding *Meriones* rats an HCD during 15 weeks increased (*p*  < 0.001) the final body weight (228.00 ± 6.81 g), fasting blood glucose (259.00 ± 12.72 mg/dl), heartbeat (327.00 ± 11.31 nb/min), and systolic arterial pressure (154.00 ± 2.12 mm Hg) as compared to control *Meriones* fed the standard chow (360 kcal/day/100 g body weight).

The oral administration of the CV extract to normal rats (NC + CV) decreased the animals' weight significantly as compared to normal and untreated diabetic *Meriones* (171.75 vs. 138.25 g and 171.75 vs. 228.00 g, respectively). However, CV extract administration did not change the normal values of other investigated parameters. Moreover, the daily treatment with the CV extract (HCD/sls + CV) as well as the simultaneous coadministration of amlodipine and glibenclamide for 3 consecutive weeks reduced the final body weight, FBG, heartbeat, and systolic arterial pressure as compared to the nontreated diabetic group.

#### 3.2.3. Urinary Determinations of Renal Biomarkers

*(1) Urinary Creatinine, Urea, and Urinary Volume*.  Table 4 represents the urinary concentrations of creatinine, urea, and urinary volume in both treated and untreated diabetic animals. The HCD/SLS group showed a significant decrease in creatinuria (*p*  < 0.05), urinary urea (*p*  < 0.01), and urinary outflow (*p*  < 0.001) as compared to nondiabetic groups (NC + DW and NC + CV). However, the daily treatment of the HCD/SLS group with the CV extract (group 4) for 3 consecutive weeks resulted in a significant increase of both the urinary urea level and urine output in comparison to the baseline (day 0) as well as in comparison to the diabetic nontreated group (HCD/SLS + DW). Present results also indicate that the combined cotreatment (HCD/SLS + Aml + *G*) leads to a significant increase in urinary urea and creatinuria levels. On the opposite, it highly decreased (*p*  < 0.001) the urinary volume as compared to all experimental groups.

*(2) Urinary Electrolytes (Na^+^, K^+^, and Cl^−^)*. Regarding urinary electrolytes, the data in Table 5 showed that HCD-fed rats displayed high urinary leakage of sodium, potassium, and chloride (*p*  < 0.001) in comparison to normal *Meriones shawi* rats (group 2). On the opposite, the simultaneous cotreatment by Aml + *G* (group 5) significantly reduced urinary electrolytes (Na^+^, K^+^, and Cl^−^) as compared to *Meriones* fed the standard chow diet. Diabetic *Meriones* treated with the CV extract for 3 consecutive weeks expressed high leakage of all investigated urinary electrolytes (*p*  < 0.001) as compared to all groups. In contrast, the HCD/SLS group treated simultaneously with amlodipine and glibenclamide HCD/SLS + (Aml + *G*) (group 5) showed a significant decrease in all urinary electrolytes as compared to untreated and treated diabetic groups (HCD/SLS + DW and HCD/SLS + CV).

### 3.3. Vascular Reactivity

#### 3.3.1. Ex Vivo Effect of CV Treatment on the Contractile Responses to Noradrenaline

The effect of CV treatment on normal and high-calorie diet/sedentary lifestyle (HCD-SLS) *Meriones shawi* rats was evaluated *ex vivo* using isolated aortic rings.

**Figure 1:**
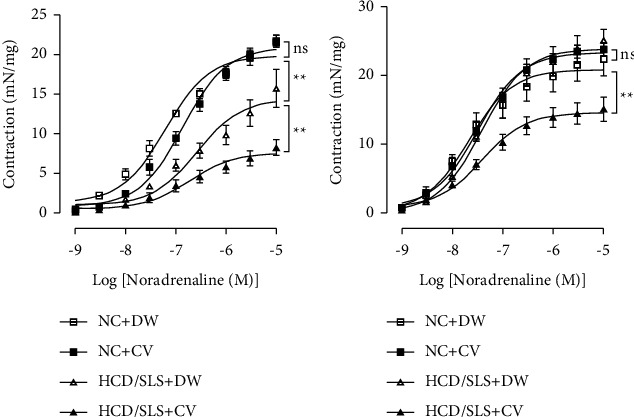
*Ex vivo* effect of the *Calycotome vilosa* extract on noradrenaline concentration-response curves in aorta from lean and diabetic *Meriones* in the absence (a) or in the presence of L-NOArg (b) Each value is the mean of 6 determinations. Significant difference between control and treated groups was performed at high concentration and was indicated as follows: ^*∗∗*^*p*  < 0.01 and ^*∗∗∗*^*p*  < 0.001.

To explore whether the oral administration of the CV extract possesses an effect on the reactivity of diabetic-hypertensive *Meriones shawi* rats aorta, noradrenaline (Nad) (1 nM–10 *μ*M)-induced contractile response curves were performed without (Figure 1(a)) and with (Figure 1(b)) NO synthase inhibitor L-NG-nitro arginine (L-NOArg, 100 *μ*M).

In the absence of L-NOArg (Figure 1(a)), the noradrenaline concentration-response curve was significantly depressed in diabetic *Meriones* as compared to normal controls with a maximum effect reaching 15.73 Vs. 8.26 mN/mg for diabetic and normal *Merions*, respectively (*p* < 0.01), whereas, the addition of L-NOArg markedly increased contractions in diabetic meriones compared to normal *Meriones*. Contraction in response to noradrenaline (10 *μ*M) reached 15.09 ± 1.75 mN/mg vs. 25.09 ± 1.61 mN/mg for normal and diabetic *Meriones*, respectively (Figure 1(b)).

CV treatment has no significant effect on the noradrenaline contractile responsecurve of control *Meriones*, but a slight inhibition and a slight increase were observed in the absence and the presence of L-NOArg, respectively. In both experimental conditions (the presence or the absence of L-NOArg), the contractile responses of aortic rings from CV-treated diabetic *Meriones* were significantly depressed compared to those of untreated-diabetic *Meriones* arteries.

In the presence of L-NOArg, the Emax-Nad was reduced by 39.75% as compared to the untreated HCD/SLS group, precisely from 25.09 to 15.09 mN/mg. However, in the absence of L-NOArg, the Emax − Nad was reduced by 47.429% from 15.73 to 8.26 mN/mg (Table 6). Indeed, the effect of the CV extract on Nad Emax was higher pronounced in the absence than in the absence of L-NOArg. The pEC50 value of Nad was not significantly changed between the untreated diabetic group and the CV-treated diabetic group, neither in the presence nor in the absence of L-NOArg (Table 6).

#### 3.3.2. Ex Vivo Effect of CV Treatment on Endothelium-Dependent Relaxation

**Figure 2:**
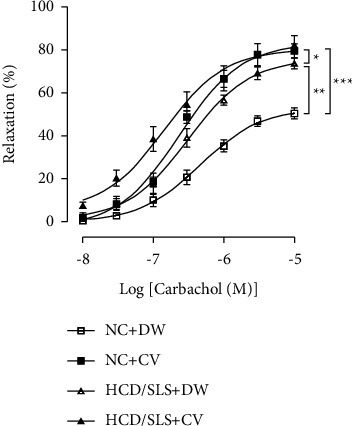
*Ex vivo* effect of *Calycotome vilosa* treatment on endothelium-dependent relaxation evoked by carbachol in noradrenaline-precontracted aorta from control and diabetic *Meriones*. Values are expressed as a percentage of the precontraction level and represent means ± SEM of 6 determinations. Significant difference between control and treated groups was performed at a high concentration and was indicated as follows: ^*∗*^*p*  < 0.05, ^*∗∗*^*p*  < 0.01, and ^*∗∗∗*^*p*  < 0.001.

To determine whether the daily oral administration of the CV extract (50 mg/kg.bwt) can affect the endothelium-dependent relaxation induced by carbachol, *ex vivo* investigations were performed in aortic rings isolated from each rat of all experimental groups (Figure 2). In endothelium-intact vessels, the addition of carbachol to noradrenaline-precontracted aorta produced a larger relaxation of aorta rings from HCD/SLS *Meriones* compared to normal animals (NC + DW) (*p* < 0.01). Emax were 73.43 ±± 2.40 and 50.21 ± 2.57, respectively. However, pD_2_ values were not significantly changed between normal and HCD/SLS *Meriones* (6.33 ± 0.18 vs. 6.50 ± 0.17). As compared to untreated HCD/SLS *Meriones*, Nad precontracted aortas from HCD/SLS treated *Meriones* (HCD/SLS + CV) exhibited slight relaxation in response to carbachol (*p* < 0.05). Emax were 73.43 ± 2.40 and 82.10 ± 4.15, respectively. However, similar concentration-response curves have been documented compared to the normal treated group (NC + CV).

#### 3.3.3. Ex Vivo Effect of CV Treatment on Endothelium-Independent Relaxation

**Figure 3:**
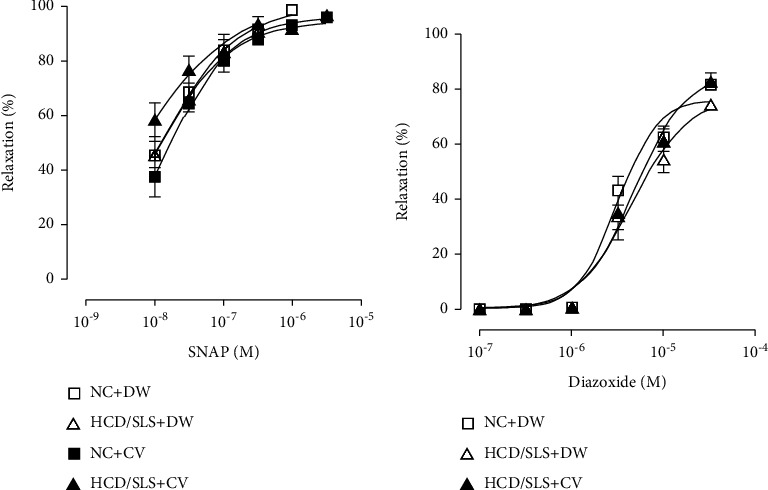
*Ex vivo* effect of *Calycotome vilosa* treatment on endothelium-independent relaxation evoked by SNAP (a) or diazoxide (b) in noradrenaline-precontracted aorta from control and diabetic *Meriones*. Values are expressed as a percentage of the precontraction level and represent means ± SEM of 6 determinations.

To ascertain whether daily administration of the CV extract for 3 consecutive weeks can influence the endothelium-independent relaxation induced by SNAP (Figure 3(a)) or by diazoxide (Figure 3(b)), experiments were performed in aortic rings isolated from *Meriones* of all groups. As shown in Figure 3, the vasorelaxation response evoked by SNAP or diazoxide in the noradrenaline-precontracted aorta was not significantly affected neither by HCD nor by CV treatment.

### 3.4. Effect of CV Treatment on Aorta Relaxation in Response to L-Arginine or Insulin

**Figure 4:**
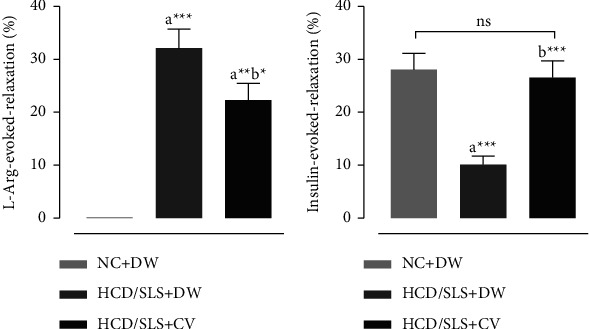
Relaxation of noradrenaline contraction in response to L-arginine (a) or insulin (b) in aorta from control and untreated and *Calycotome vilosa*-treated diabetic *Meriones*. Aorta was precontracted with noradrenaline (1 *μ*M) before the addition of L-arginine (100 *μ*M) or insulin (50 *μ*U/ml). Values are expressed as a percentage of the precontraction and represent means ± SEM of 6 determinations. Significant difference between normal and treated groups was performed at a high concentration and was indicated as follows: ^*∗*^*p*  < 0.05, ^*∗∗*^*p*  < 0.01, and ^*∗∗∗*^*p*  < 0.001.

The vasorelaxation effect evoked by insulin (Figure 4(b)) was significantly lowered in HCD/SLS *Meriones* compared to *Meriones* fed the standard chow (NC + DW) (*p* < 0.01). However, it is markedly increased following CV extract treatment (HCD/SLS + CV). On the opposite, in response to L-arginine (Figure 4(a)), aorta from animals fed HCD and submitted to limited physical inactivity showed a significant vasorelaxation effect as compared to the aorta from normal *Meriones*. More importantly, the CV extract decreased L-arginine evoked relaxation by 30.07%, from 31.95% to 22.34%.

## 4. Discussion

The present study documented that a high-calorie diet (HCD) (612 kcal/day/100 g body weight) associated with a sedentary lifestyle (SLS) (physical inactivity) leads to hyperglycemia, insulin resistance, obesity, and hypertension associated with vascular reactivity alteration.

Obtained results go in hand with the finding of our previous data [1] and support the results of Ferreira-Santos et al. who reported that the administration of a high fructose-enriched diet (20%) to rats for 12 weeks induced hypertriglyceridemia, hyperglycemia, hypertension, oxidative stress, and endothelial dysfunction, which are the hallmarks and common disorder of metabolic syndrome [19]. In addition to the resulting imbalance between energy intake and expenditure, HCD induces the metabolic syndrome through the over-liberation of free fatty acids in various organs [20, 21]. Nowadays, the sedentary lifestyle associated with a high-calorie diet is considered as a key risk factor involved in the development of metabolic disorders, especially T2D, obesity, and high blood pressure [22]. The chronic HCD intake (15 weeks) significantly increased the body weight, fasting blood glucose, heartbeat, and systolic arterial pressure as compared to nontreated normal *Meriones* (NC + DW), which is in accordance with the data of Nascimento and coworkers, reporting that hypercaloric pellet-diet administration over 14 weeks induced diabesity and promoted a remarkable increase in the systolic arterial pressure as compared to control rats [23]. Most importantly, normal *Meriones* treated with the CV extract (NC + CV) expressed a significant decrease in the final body weight and showed normal values of other evaluated parameters, which illustrates the catabolism activity and the safety of the CV extract at the tested dose (50 mg/kg.bwt). However, the daily oral administration of the CV extract to diabetic *Meriones* at the same dose as well as the simultaneous coadministration of amlodipine and glibenclamide for 3 weeks reduced the FBG, heartbeat, and systolic arterial pressure as compared to the nontreated diabetic group (HCD/SLS). These results suggest that the CV extract might improve fasting blood glycemia by normalizing the postprandial plasma glucose level, which is supported by the OGTT data (Table 2).

It has been documented that HCD intake induces hyperglycemia and oxidative stress through the overproduction of reactive oxygen species (ROS) and the activation of different inflammatory pathways [18, 24]. In fact, the overproduced ROS inhibits insulin signaling and consequently, causes its resistance by peripheral tissues [25, 26]. To our knowledge, till now, no document has reported the hypoglycemic or antidiabetic effect of CV. However, several studies have confirmed that terpenoids and flavonoids among other antioxidant molecules are the main bioactive compounds involved in the hypoglycemic activity of numerous medicinal and aromatic plants (almost 1200 plants) [27, 28].

Although several plants have been traditionally used to treat obesity, diabetes, and their complications, only a few plants have been experimentally studied and even fewer are being scrutinized for the mechanisms responsible for their antidiabesity effects.

*Calycotome villosa* Subsp*. Intermedia* is a popular medicinal plant used in Moroccan traditional medicine for the prevention and self-treatment of several health disorders, including diabetes mellitus, obesity, and hypertension. However, so far, this is the first study designed to assess the potential hypoglycemic and hypotensive effects of the CV extract to confirm its traditional use.

*Calycotome Villosa* Subsp*. Intermedia* is an inexhaustible source of bioactive molecules [29]. Its antidiabetic potential may be related to chemical interactions that occurred among their individual effective components through one or several signaling pathways. Indeed, the hypoglycemic effect of the CV extract could be due to the following: (a) its inhibitory effect of glucose-6 phosphatase and fructose 1, 6 biphosphatase activities such as revealed by [30] for the *Acorus calamus* rhizome extract; (b) its insulin-stimulatory effect accompanied by the glucose intestinal inhibitory absorption effect such as shown for leaves' hydroalcoholic extract of *Adina cordifolia* [31]; and (c) potentiation of insulin synthesis from *β* cells and promotes peripheral absorption of glucose, as indicated for the stem bark extract *of Afzelia africana* [32]. In a recent study, Ansari and coworkers investigated the effect of the hot water extract of 22 medicinal plants used traditionally to treat diabetes on dipeptidyl peptidase-IV (DPP-IV) activity both in vitro and in vivo in high-fat fed (HFF) obese-diabetic rats. Results showed that all examined extracts displayed DPP-IV inhibitory effects ranging between 9 and 96% [33]. This could be one of the most possible mechanisms of action by which CV ensures its antidiabetic effect.

Chronic hyperglycemia represents the main cause of diabetes complications, and it is considered a key factor associated with morbidity and mortality worldwide, especially when it is uncontrolled [34]. Diabetic nephropathy is one of these serious complications [35]. Creatinine is a normal metabolic waste produced by the body from muscle creatinine, while urea is a nitrogenous waste product that is broken down by the liver from proteins, then filtered by the kidneys, and excreted by the urine [36]. Chronic hyperglycemia may cause dehydration and deficiency of cations through the development of osmotic diuresis. Our data indicate that HCD intake decreased creatinuria, urinary urea, and urinary outflow, which eventually leads to their accumulation in the blood and subsequently induces diabetic nephropathy [37]. These results are in accordance with the finding of Akinnuga et al. who reported that the supplementation of high-fat high-carbohydrate (HFHC) for 20 weeks to normal male Sprague–Dawley rats resulted in a significant imbalance of renal biomarkers characterized by a remarkable decrease in urinary creatinine, urea, and glomerular filtration rate [38].

CV treatment (HCD/SLS + CV) significantly increased both the urinary urea level and urine output in comparison to the first week of treatment as well as in comparison to the diabetic nontreated animals. Therefore, it reduces the blood waste products and thus, prevents the reno-toxicity and kidney damage induced by the hyper-calorie diet. Our data are in agreement with those of Snehal and coworkers who reported that the *Punica granatum* Linn. leaves extract improves diabetic nephropathy in streptozotocin-induced type 1 diabetes (T1D) in rats [39].

Due to their important role in the regulation of blood pressure, the assessment of electrolyte balance is an essential step in the diagnosis of cardiometabolic diseases, especially T2D and hypertension [40]. Normal *Meriones* submitted to high-calorie diet (HCD/SLS + DW) expressed a high urinary leakage of sodium, potassium, and chloride as compared to normal rats (NC + DW). Likewise, increased urinary excretion of sodium, potassium, and chloride ions has been also documented by Chen et al. in streptozotocin-induced type 1 diabetes [41] and by Eteng et al. who reported that electrolytes loss in uncontrolled diabetes mellitus is most often related to osmotic diuresis [42]. However, CV extract treatment significantly increased the leakage of all investigated urinary electrolytes, which is most probably referred to its detoxifying charge in addition to its hypotensive proprieties [14]. These results are in agreement with our previous study in which we demonstrated the diuretic effect and the potent electrolyte excretion capacity of chrysin glucoside isolated from CV [14]. Accordingly, we documented that the continuous perfusion of chrysin glucoside extracted from different parts of *Calycotome villosa* Subsp *Intermedia* at a dose of 2.5 mg/kg significantly increased the electrolyte excretion (Na^+^ and K^+^), urine flow, and glomerular filtration (*p* < 0.001). Similarly, other studies have associated the diuretic effect of herbal plants with the activity of their secondary metabolites such as saponins through their ability to modulate the membrane permeability and stimulate the renal excretion of sodium [43, 44]. In addition, flavonoids have been found to exhibit urinary output with a potent natriuresis and kaliuresis charge [45]. In this context, various phytochemical investigations have shown the presence of several metabolites such as flavonoids, polyphenols, alkaloids, and saponins in different extracts of pods, seeds, leaves, and flowers of *Calycotome villosa* Subsp *Intermedia* [15, 46, 47]. Therefore, the documented urinary excretion following the subchronic treatment by the CV extract could be attributed to the presence of saponins and flavonoids as well as to the presence of other bioactive phytoconstituents.

As documented in our previous investigation [1], the relaxation evoked by carbachol was significantly increased in HCD/SLS *Meriones* as compared to normal *Meriones* (NC + DW). These results contrast with the finding of previous data describing the reduction of endothelium-dependent relaxation of T2D patients [48] and T2D/hypertensive rats [49]. In general, the impaired vascular relaxation could be attributed to one or multiple mechanisms of action, including the decrease of NO sensitivity/exogenous NO evoked relaxation and/or the alteration of the NO/cyclic guanosine monophosphate (cGMP) pathway [50]. Recently, Enging and coworkers pointed out that phosphorylated vasodilator-stimulated phosphoprotein p-VASP^Ser239^/total VASP and p-eNOS^Ser1177^/total eNOS ratios were significantly lower in the diabetic rats' aortas compared to normal rats' aorta, indicating that diabetes prompts vascular smooth muscle impairment and endothelial dysfunction throughout the reduction of eNOS activity and the downregulation of the NO/cGMP signaling pathway [51]. In contrast, other reports showed that chronic hyperglycemia stimulates eNOS and iNOS gene expression and enhances NO production [52, 53].

Till now, the relationship between diabetes and NO production remains controversial. This contradiction may seem illogical, but many researchers pointed out that the animal's age and the severity level of other cardiometabolic complications influence the NO production and, thus, the endothelium-dependent vasorelaxation. In this sense, Adela and coworkers investigated the association between chronic hyperglycemia and serum NO levels in T2DM patients with and without cardiovascular complications (hypertension). Authors reported that the serum NO level was significantly higher in T2DM without hypertension as compared to T2DM patients with cardiovascular complications (111.8 *μ*M vs. 68.2 *μ*M, respectively) and significantly higher in T2DM patients suffering from diabetes of less than 5 years compared to diabetic subjects more than 5 years (115.3 and 83.5 *μ*M, respectively). [52]. Additionally, it has been documented thirty years ago that nitric oxide levels and NO-mediated endothelium-dependent relaxation depend on the stage of diabetes mellitus.

The vasorelaxation response evoked by SNAP or diazoxide (endothelium-independent) in the noradrenaline-precontracted aorta was not significantly changed between rings obtained from both treated and untreated HCD/SLS *Meriones*. Owing to its ability to release NO within the vascular smooth muscle cells immediately after its metabolism, most probably, these pathways are not implicated in the impairment of endothelium-dependent vasodilatation induced by HCD/SLS. Once released from the SNAP, NO binds typically to the soluble guanylate cyclase (sGC) and thus enhances the cyclic guanosine-3′,5′-monophosphate (cGMP) generation, leading to aorta vasodilation [54]. In this regard, the present results indicate that the high-calorie diet associated with a sedentary lifestyle as well as *Calycotome villosa* treatment did not affect the sensitivity and the smooth muscle response to NO.

Regarding vascular reactivity, the oral administration of the CV extract did not markedly modify the noradrenaline contractile response-curve in control *Meriones*. However, a slight inhibition and a slight increase were exhibited in the absence and the presence of L-NOArg, respectively. Moreover, it markedly depressed contractions in diabetic *Meriones* in both experimental conditions. These results were consistent with those of El-Hilaly et al. who reported that the short-term treatment with *Ajuga iva* extracts to SHR-SP rats induced a significant decrease of aortic ring contraction in the presence and in the absence of noradrenaline compared to normal untreated animals [16].

In response to carbachol, CV treatment exhibited a significant NO-dependent vasorelaxation effect in healthy and diabetic aorta *Meriones*, with the best effect observed in normal *Meriones* (NC + CV). Since oxidative stress and inflammation are involved in the mechanism of endothelial dysfunction and the pathophysiology of T2D associated with hypertension, the monitored hypotensive and vasorelaxant effect of the CV extract could be due to its rich composition on bio-valuable antioxidant and anti-inflammatory molecules. Previously published data reported that *Calycotome villosa* seed extracts contain several natural antioxidants belonging to different chemical groups such as flavonol, flavone, flavanone, flavan‐3‐ol, hydroxycinnamic acids, and hydroxybenzoic acids [15]. According to this study, quercetin, rutin, kaempferol, luteolin, apigenin, naringenin, epicatechin, p‐coumaric acid, syringic acid, and quinic acid were the most predominant quantified phenolic compounds. In addition, these compounds are also present in other medicinal plants and functional food products known for their antioxidative, antidiabetic, and antihypertensive properties [16, 17].

Recent literature data reported the contribution of oxidative stress in the pathophysiology of cardiovascular dysfunction and pointed out that quercetin mediates this cardioprotective effect by balancing ROS and other free radicals production, inhibiting ROS-dependent signaling pathways, suppressing inflammation-mediated damage, upregulating nonantioxidant enzymes, enhancing antioxidant enzyme activities, and by upgrading mitochondria function [55]. In the same context, the outcome of Ren et al. showed that apigenin improves fasting blood glucose level, insulin sensitivity, vascular dysfunction, and controls lipid metabolism in T2D rats [56]. In addition, apigenin-induced endothelium-dependent vasodilation in rats' aortic rings by minimizing oxidative stress and reinstating the reduced NO [57]. Moreover, rutin, a flavonol present in CV extracts, has been documented to prevent hypertension and vascular complication by modulating NOX4 and ROS-sensitive NLRP3 inflammasome as a result of its antioxidant and anti-inflammatory action [58].

Owing to its effect on vascular contractility, insulin plays a pivotal role in maintaining metabolic and hemodynamic homeostasis. It has been found that insulin-evoked vasorelaxation in both animal and human models by promoting endothelium-dependent NO production [59]. The relaxation effect (%) evoked by insulin was markedly reduced in HCD/SLS *Meriones* compared to normal *Meriones* (NC + DW) or (NC + CV), which might be attributed to insulin resistance [1]. Generally, in insulin-resistant states, chronic hyperglycemia downregulates the endothelial nitric oxide synthase (eNOS) and inhibits its phosphorylation, thus impairing the NO-mediated endothelium-dependent relaxation/dilatation [60]. In CV-treated diabetic *Meriones* (HCD/SLS + CV), insulin improved the endothelium-dependent relaxation. Obtained results are in agreement with those published by Kobayashi and coworkers, in which insulin upregulates the mRNA expression of NOS, leading to NO generation and restoring the endothelial-dependant relaxation in aortae from T2D rats [61].

Endothelial dysfunction has been suggested as a common disorder linking hyperglycemia, hyperinsulinemia, insulin resistance, and hypertension [19]. Substantial evidence specifies that flavonoids and nonflavonoids components (terpenoids, phenolic acids, stilbenes, and microantioxydant nutrients) act together and control endothelial dysfunction risk factors by improving lipid and glucose metabolism, as well as insulin resistance [62, 63]. This supports the hypothesis of their contribution to the mechanism of insulin sensitivity management, hypotensive effect, and the improvement of endothelial dysfunction. Despite these encouraging results, this study is still preliminary; an entirely chemical characterization and other complementary experiments will be necessary to draw the necessary recommendations to secure its traditional use.

Overall, the hypoglycemic and hypotensive effects of the CV extract could be due to binary or multiple interactions among its bioactive components through one or diverse signaling pathways.

## 5. Conclusion

Results of the present study showed for the first time that the CV extract improves hyperglycemia and hypertension induced by a high-calorie diet associated with a sedentary lifestyle. The role of this extract may involve the improvement of fasting blood glucose, insulin resistance, electrolyte balance, and urinary volume output. Furthermore, this extract decreases vascular contraction to noradrenaline. These effects may be due, at least in part, to phenolic and no phenolic bioactive ingredients contained in the CV extract. These findings support the traditional use of CV and provide a basic approach for the management of T2D and hypertension-related vascular dysfunction and possibly can arise as a coadvantageous strategy to reduce the incidence of cardiovascular risk factors and cardiometabolic diseases. Further studies are required to investigate the precise mechanism of action by which the CV extract, possibly antioxidant components, enhance Mets, leading to the identification of potential antidiabetic and antihypertensive agents for future drug development [64].

## Figures and Tables

**Table 1:** Effect of sedentary lifestyle associated with the high-calorie diet for 12 weeks on fasting blood glucose, total cholesterol, triglycerides, and body weight.

Parameters	Treatment duration (weeks)						
	0	2	4	6	8	10	12
Blood glucose (mg/dL)	76.00 ± 4.53	92.00 ± 5.65	99.10 ± 4.36	148.50 ± 7.77	175.50 ± 6.36^a^^*∗*^	198.00 ± 5.65^a^^*∗*^	222.00 ± 7.0^a^^*∗∗*^
Total cholesterol (mmol/L)	1.21 ± 0.06	1.56 ± 0.02^b^^*∗∗*^	1.86 ± 0.03^b^^*∗∗∗*^	2.21 ± 0.03^b^^*∗∗∗*^	2.49 ± 0.05^b^^*∗∗∗*^	2.95 ± 0.08^b^^*∗∗∗*^	3.19 ± 0.06^b^^*∗∗∗*^
Triglycerides (mmol/L)	0.58 ± 0.04	0.60 ± 0.02	0.69 ± 0.03	0.79 ± 0.05^c^^*∗*^	1.11 ± 0.04^c^^*∗∗∗*^	1.24 ± 0.06^c^^*∗∗∗*^	1.39 ± 0.08^c^^*∗∗∗*^
Body weight (g)	103 ± 7.15	n.p	145 ± 10.33^d^^*∗*^	n.p	182 ± 8.16^d^^*∗∗*^	n.p	219 ± 8.5^d^^*∗∗∗*^

a: comparison of blood glucose level between week 0 and all weeks, b: comparison of total cholesterol between week 0 and all weeks, c: comparison of triglycerides between week 0 and all weeks, d: comparison of body weight between week 0 and all weeks (^*∗*^*p*  < 0.05, ^*∗∗*^*p*  < 0.01, and ^*∗∗∗*^*p*  < 0.001). n.p: not performed.

**Table 2:** Blood glucose level changes during 180 minutes after a single oral administration of the CV extract in normal and treated animals.

Experimental groups	Blood glucose levels (mg/dl)-time (minutes)							
	−30	0	15	30	45	60	120	180
HCD/SLS+DW	203.00 ± 5.65^b^^*∗∗∗*^^c^^*∗∗∗*^	202.00 ± 7.1^b^^*∗∗∗*^^c^^*∗∗∗*^	254.00 ± 5.38^a^^*∗∗*^^b^^*∗∗∗*^^c^^*∗∗∗*^	271.50 ± 6.1^a^^*∗∗∗*^^b^^*∗∗∗*^^c^^*∗∗∗*^	259.00 ± 7.1^a^^*∗∗∗*^^b^^*∗∗∗*^^c^^*∗∗∗*^	244.00 ± 7.4^a^^*∗∗*^^b^^*∗∗∗*^^c^^*∗∗∗*^	236.00 ± 8.5^a^^*∗∗*^^b^^*∗∗∗*^^c^^*∗∗∗*^	229.00 ± 10.6^a^^*∗*^^b^^*∗∗∗*^^c^^*∗∗∗*^
NC + DW	85.00 ± 2.8^c^^*∗*^	83.00 ± 4.2^c^^*∗*^	146.00 ± 7.1^a^^*∗∗∗*^	202 ± 4.2^a^^*∗∗∗*^^c^^*∗*^	150.00 ± 5.6^a^^*∗∗∗*^	116 ± 5.3^a^^*∗∗*^^c^^*∗*^	89.00 ± 4.2^c^^*∗∗*^	84.00 ± 5.6^c^^*∗∗*^
NC + CV	85.00 ± 4.2	82.00 ± 5.4	130.00 ± 7.1^a^^*∗∗∗*^	169.00 ± 5.6^a^^*∗∗∗*^^b^^*∗*^	147.00 ± 8.5^a^^*∗∗∗*^	114.00 ± 7.1^a^^*∗∗*^	87.00 ± 4.2	84.00 ± 6.9
HCD/SLS + CV	213.00 ± 5.6^b^^*∗∗∗*^	207.00 ± 8.1^b^^*∗∗∗*^	152.00 ± 7.3	175.00 ± 7.1^a^^*∗∗*^^b^^*∗*^	157.00 ± 8.2^a^^*∗∗∗*^	148.00 ± 5.8^a^^*∗∗∗*^^b^^*∗*^	138.00 ± 5.6^a^^*∗∗*^^b^^*∗∗*^	134.00 ± 3.1^a^^*∗*^^b^^*∗∗*^

a: as compared to blood glucose level at 0 time, b: in comparison to normal groups, and c: in comparison to diabetic treated groups.

**Table 3:** Effect of the CV extract on blood glucose, heartbeat, and systolic arterial pressure in normal and treated animals.

Parameters	Treatment duration (weeks)	Experimental groups				
		HCD/SLS + DW	NC + DW	NC + CV	HCD/SLS + CV	HCD/SLS + (Aml + *G*)
Body weight (g)	0	221.00 ± 8.17	160.37 ± 2.60	171.75 ± 2.13	222.9 ± 6.83	225.00 ± 10.40
	1	221.60 ± 6.97	156.25 ± 4.01	147.75 ± 2.28	202.3 ± 6.20	223.33 ± 8.81
	2	222.40 ± 6.33	159.00 ± 2.92	143.50 ± 2.39^a*∗*^	182.1 ± 5.03^a*∗*^	221.66 ± 9.28
	3	228.00 ± 6.81^b*∗∗∗*^	157.50 ± 3.17	138.25 ± 1.65^a*∗∗*^	166.9 ± 4.99a^*∗∗*c*∗∗∗*^	222.60 ± 10.26^b*∗∗∗*^

Blood glucose (mg/dl)	0	220.00 ± 5.42	85.10 ± 4.10	84.18 ± 4.15	218.82 ± 6.10	228.10 ± 8.34
	1	228.00 ± 9.82	83.75 ± 5.30	85.29 ± 4.73	186.50 ± 6.36^a*∗*^	219.40 ± 6.22
	2	235.00 ± 9.89	87.60 ± 4.80	85.85 ± 4.45	139.50 ± 10.60^a*∗∗*^	201.50 ± 6.36^a*∗*^
	3	259.00 ± 12.72^a*∗*b*∗∗∗*^	84.75 ± 6.43	83.50 ± 4.49	120.00 ± 5.65^a*∗∗∗*b*∗*c*∗∗∗*^	154.00 ± 8.48^a*∗∗∗*b*∗∗*c*∗∗∗*^

Heart beat (nb/min)	0	330.00 ± 17.32	270.00 ± 0.00	300.00 ± 0.00	315.00 ± 15.00	300.00 ± 00.00
	1	322.50 ± 22.50	270.00 ± 0.00	292.50 ± 7.50	307.50 ± 18.87	300.00 ± 00.00
	2	319.00 ± 14.14	270.00 ± 0.00	285.00 ± 8.66	285.00 ± 8.66	280.00 ± 6.66
	3	325.00 ± 15.00^b*∗*^	290.00 ± 8.66	287.50 ± 14^c*∗*^	277.50 ± 14.36^a*∗*c*∗*^	270.00 ± 15.00^a*∗*c*∗*^

SAP (mm Hg)	0	152.00 ± 4.24	106.00 ± 2.82	110.00 ± 4.12	149.00 ± 7.07	105.50 4.94
	1	155.50 ± 3.53	105.50 ± 3.13	108.00 ± 3.56	145.00 ± 5.65	127.00 ± 5.65^a*∗*^
	2	151.00 ± 5.65	104.50 ± 5.32	106.50 ± 5.41	121.5.00 ± 4.94^a*∗*^	151.00 ± 7.18^a*∗∗*^
	3	154.00 ± 2.12^b*∗∗∗*^	107.00 ± 4.16	106.00 ± 4.73	102.00 ± 6.33^a*∗∗*c*∗∗∗*^	105.50 ± 3.53^a*∗∗*c*∗∗∗*^

a: comparison of variables between week 0 and all weeks, b: comparison between the normal group and all groups at week 3, c: comparison between the diabetic group (DC) and the treated diabetic group at week 3 (^*∗*^*p*  < 0.05, ^*∗∗*^*p*  < 0.01, and ^*∗∗∗*^*p*  < 0.001).

**Table 4:** Effect of daily oral administration of the CV extract on creatinine, urea, and urinary volume in normal and treated animals.

Parameters	Treatment duration (weeks)	Experimental groups				
		HCD/SLS + DW	NC + DW	NC + CV	HCD/SLS + CV	HCD/SLS + (Aml + *G*)
Creatinine (mg/dL)	0	78.73 ± 0.21	79.98 ± 0.24	78.58 ± 0.21	76.92 ± 0.28	100.97 ± 0.13
	1	74.53 ± 0.14	78.88 ± 0.19	75.94 ± 0.35	78.34 ± 0.09	110.52 ± 0.10
	2	80.76 ± 0.28	78.64 ± 0.07	80.37 ± 0.63	92.16 ± 0.16	119.51 ± 0.18^a*∗*^
	3	74.44 ± 0.28^b*∗*^	84.22 ± 0.10	83.25 ± 0.42	111.65 ± 0.19^a*∗∗∗*b*∗∗∗* c*∗∗*^	146.45 ± 0.11^a*∗∗* b*∗∗∗* c*∗∗∗*^

Urea (mg/dL)	0	1171.06 ± 5.65	1124.88 ± 4.24	1122.12 ± 4.94	1201.45 ± 4.42	1096.63 ± 7.16
	1	1061.07 ± 7.77	1107.56 ± 7.62	1145.32 ± 3.53	1247.42 ± 5.65	1597.30 ± 6.36^a*∗∗*^
	2	1120.41 ± 4.24	1140.99 ± 2.82	1158.28 ± 6.18	1360.03 ± 13.43^a*∗∗*^	1711.29 ± 5.72^a*∗∗∗*^
	3	1009.03 ± 6.36^b*∗∗*^	1083.66 ± 8.48	1142.00 ± 4.24^b*∗*^	1432.67 ± 7.77^a*∗∗*b*∗∗∗*c*∗∗*^	1864.03 ± 9.19^a*∗∗∗* b*∗∗∗*c*∗∗∗*^

UV (mL/dL)	0	8.71 ± 0.04	12.19 ± 0.28	12.61 ± 0.12	8.24 ± 0.13	9.21 ± 0.11
	1	9.66 ± 0.09^a*∗∗*^	12.55 ± 0.10	12.18 ± 0.07	9.70 ± 0.14^a*∗∗*^	8.17 ± 0.24^a*∗∗*^
	2	9.55 ± 0.17^a*∗∗*^	12.27 ± 0.21	12.13 ± 0.16	11.36 ± 0.19^a*∗∗∗*^	7.48 ± 0.13^a*∗∗*^
	3	9.61 ± 0.08^a*∗∗*b*∗∗∗*^	12.55 ± 0.14	12.41 ± 0.09	12.18 ± 0.22^a*∗∗∗*c*∗∗∗*^	6.84 ± 0.14^a*∗∗∗* b*∗∗∗*c*∗∗∗*^

a: comparison of variables between the week 0 and all weeks, b: comparison between the normal group and all groups at week 3, c: between the diabetic group and the treated diabetic group at week 3 (^*∗*^*p*  < 0.05, ^*∗∗*^*p*  < 0.01, and ^*∗∗∗*^*p*  < 0.001).

**Table 5:** Effect of the CV extract on urinary electrolyte (sodium, potassium, and chloride) levels in normal and treated animals.

Parameters	Treatment duration(weeks)	Experimental groups				
		HCD/SLS + DW	NC + DW	NC + CV	HCD/SLS + CV	HCD/SLS + (Aml + *G*)
Urinary Na^+^ (mmol/24 h)	0	4.90 ± 0.14	3.40 ± 0.13	3.44 ± 0.09	5.09 ± 0.14	3.43 ± 0.09
	1	5.05 ± 0.07	3.62 ± 0.14	3.68 ± 0.02	6.24 ± 0.16	3.66 ± 0.05^a*∗∗*^
	2	4.71 ± 0.15	3.19 ± 0.14	3.18 ± 0.12	7.02 ± 0.15	3.25 ± 0.19^a*∗∗∗*^
	3	4.95 ± 0.11^b*∗∗∗*^	3.51 ± 0.16	3.51 ± 0.08	8.93 ± 0.1^b*∗∗∗*c*∗∗∗*^	3.62 ± 0.11^a*∗∗∗*c*∗∗*^

Urinary K^+^ (mmol/24 h)	0	2.61 ± 0.09	1.44 ± 0.04	1.55 ± 0.07	2.52 ± 0.11	2.10 ± 0.12
	1	2.80 ± 0.02	1.52 ± 0.05	1.45 ± 0.02	2.90 ± 0.07^a*∗*^	2.22 ± 0.16
	2	2.46 ± 0.10	1.49 ± 0.08	1.47 ± 0.10	3.6 ± 0.09^a*∗∗∗*^	1.92 ± 0.16
	3	2.66 ± 0.09^b*∗∗∗*^	1.42 ± 0.07	1.41 ± 0.05	4.09 ± 0.09^a*∗∗∗*b*∗∗∗*c*∗∗∗*^	2.02 ± 0.18^b*∗*c*∗*^

Urinary Cl^−^(mmol/24 h)	0	5.04 ± 0.07	3.99 ± 0.07	4.00 ± 0.05	5.68 ± 0.09	3.68 ± 0.05
	1	5.22 ± 0.05	4.01 ± 0.09	3.99 ± 0.10	8.41 ± 0.07^a*∗∗∗*^	4.39 ± 0.12^a*∗*^
	2	6.39 ± 0.11^a*∗∗∗*^	4.09 ± 0.11	4.14 ± 0.02	10.89 ± 0.09^a*∗∗∗*^	4.89 ± 0.13^a*∗∗*^
	3	7.80 ± 0.09^a*∗∗∗*b*∗∗∗*^	4.02 ± 0.08	4.00 ± 0.10	12.19 ± 0.12^a*∗∗∗*b*∗∗∗*c*∗∗∗*^	5.43 ± 0.19^a*∗∗∗*b*∗∗∗*c*∗∗∗*^

a: comparison of variables between the week 0 and all weeks, b: comparison between the normal group and all groups at week 3, c: comparison between the diabetic group and the treated diabetic group at week 3 (^*∗*^*p*  < 0.05, ^*∗∗*^*p*  < 0.01, and ^*∗∗∗*^*p*  < 0.001).

**Table 6:** Effect of the CV extract on noradrenaline concentration-response in aorta from lean and diabetic meriones in the absence and in the presence of L-NOArg.

	Noradrenaline	
	*E*_max_	pD_2_
*Without L-NOArg*		
HCD/SLS + DW	15.73 ± 2.40^b*∗∗*^	6.58 ± 0.31
HCD/SLS + CV	8.26 ± 0.99^a*∗∗*^	6.72 ± 0.33
NC + DW	21.51 ± 0.20^b*∗∗∗*^	7.20 ± 0.15
NC + CV	21.71 ± 0.77^b*∗∗∗*^	6.86 ± 0.13

*With L-NOArg*		
HCD/SLS + DW	25.09 ± 1.61^b*∗∗*^	7.41 ± 0.20
HCD/SLS + CV	15.09 ± 1.75^a*∗∗*^	7.41 ± 0.28
NC + DW	22.35 ± 2.42^b*∗∗*^	7.67 ± 0.30
NC + CV	23.82 ± 0.98^b*∗∗*^	7.49 ± 0.14

a: comparison of variables between the HCD/SLS + DW group and the HCD/SLS + DC group, b: comparison between the HCD/SLS + DC group and all groups. (^*∗∗*^*p*  < 0.01 and ^*∗∗∗*^*p*  < 0.001).

## Data Availability

The data used to support the findings of this study are available from the corresponding authors upon request.
